# Prostate Cancer Dormancy and Reactivation in Bone Marrow

**DOI:** 10.3390/jcm10122648

**Published:** 2021-06-16

**Authors:** Deepak K. Singh, Vaibhav G. Patel, William K. Oh, Julio A. Aguirre-Ghiso

**Affiliations:** 1 Division of Hematology and Oncology, Department of Medicine, Department of Otolaryngology, Department of Oncological Sciences, Tisch Cancer Institute, Black Family Stem Cell Institute, Icahn School of Medicine at Mount Sinai, New York, NY 10029, USA; julio.aguirre-ghiso@mssm.edu; 2 Division of Hematology and Oncology, Department of Medicine, Tisch Cancer Institute, Icahn School of Medicine at Mount Sinai, New York, NY 10029, USA; william.oh@mssm.edu

**Keywords:** dormancy, prostate cancer, bone marrow, microenvironment, retinoic acid, azacytidine

## Abstract

Prostate cancer has a variable clinical course, ranging from curable local disease to lethal metastatic spread. Eradicating metastatic cells is a unique challenge that is rarely met with the available therapies. Thus, targeting prostate cancer cells in earlier disease states is a crucial window of opportunity. Interestingly, cancer cells migrate from their primary site during pre-cancerous and malignant phases to seed secondary organs. These cells, known as disseminated cancer cells (DCCs), may remain dormant for months or decades before activating to form metastases. Bone marrow, a dormancy-permissive site, is the major organ for housed DCCs and eventual metastases in prostate cancer. The dynamic interplay between DCCs and the primary tumor microenvironment (TME), as well as that between DCCs and the secondary organ niche, controls the conversion between states of dormancy and activation. Here, we discuss recent discoveries that have improved our understanding of dormancy signaling and the role of the TME in modulating the epigenetic reprogramming of DCCs. We offer potential strategies to target DCCs in prostate cancer.

## 1. Introduction

Cancer can recur months, years, or even decades after the initial diagnosis. It is the recurrent, metastatic form of cancer that accounts for many cancer-related deaths [1,2]. The quiet period before cancer recurrence is possible due to the cells’ ability to enter a reversible, quiescent non-proliferative state, known as cellular dormancy [3]. During the quiescent and dormant state, cancer cells are generally resistant to chemotherapies and other anti-cancer therapies, which are more effective against proliferative cells [4,5,6]. Upon pro-proliferative signaling and favorable microenvironmental cues, dormant cancer cells can exit the quiescent state and become proliferative, leading to overt metastases [7,8,9]. Cancer or cellular dormancy is different from tumor mass dormancy, which remains unchanged in tumor size due to the balance between proliferation and apoptosis of cancer cells, resulting from vascularization constraints or immune editing [10,11,12].

Prostate cancer (PCa) is a heterogeneous disease that has variable clinical outcomes ranging from early-stage, curable disease to advanced, lethal disease. Although most men are cured due to diagnosis at an early stage, a subset of men develop recurrent disease, or they present with de novo metastatic disease [13]. Several novel therapies, including androgen-receptor targeted therapies, chemotherapy, Poly ADP ribose polymerase (PARP) inhibitors, sipuleucel-T, and radium-223, are approved for advanced PCa; however, this disease still accounts for the second highest number of cancer-related deaths in men in the United States [14]. The challenges in treating this advanced state include, but are not limited to, high genomic heterogeneity [15], pro-immunosuppressive environment [16], and several emerging mechanisms of androgen independence [17]. Given that PCa can recur months, years, or decades after initial diagnosis and treatment, understanding the role of dormancy has major diagnostic and therapeutic implications. In this mini-review, we discuss mechanisms of prostate cancer dormancy and metastasis, with relevant models and data of PCa and other cancers. We propose that it is useful to draw information from other cancers that metastasize to the bone or from those that never grow there despite disseminated cancer cells (DCCs) being present. This can be used to provide insight into how to target prostate cancer metastasis initiation and maintenance.

## 2. Dormancy in Prostate Cancer

**Figure 1:**
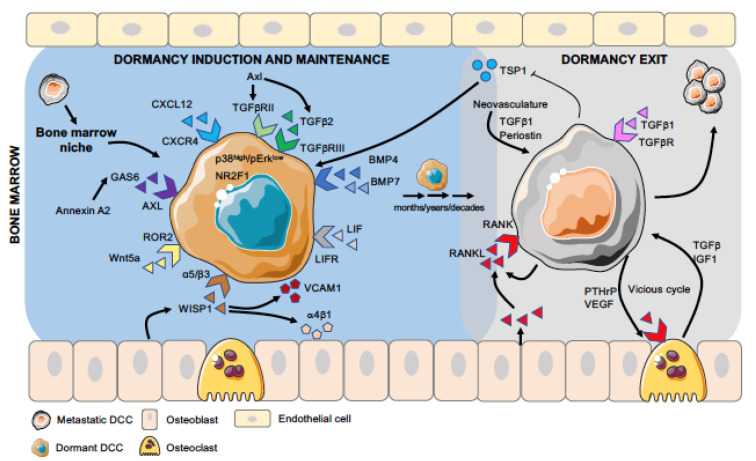
Different stages of DCC dormancy and activation in bone marrow. Disseminated cancer cells (DCCs), after arriving in the bone marrow, encounter different niches (e.g., osteogenic, peri-sinusoidal, and peri-arteriolar), where their interaction with specific cell types and extracellular matrix (ECM) molecules influence their fate. Dormancy-inducing factors, such as TGFβ2, Wnt5a, and BMP4/7, activate the dormancy program in DCCs (induction stage), and many of these factors are also involved in the maintenance of the dormant phenotype, which lasts from months to decades (maintenance stage). Pro-metastatic cues, such as RANKL expressed by PCa cells or aging osteoblasts, signal dormant DCCs to exit this state and promote proliferation and overt-metastasis (exit stage), which is further favored by the “vicious cycle” involving osteoclasts and cancer cells.

The bone is the preferred site for PCa metastasis, as >90% of patients with advanced disease develop osteoblastic bone metastases [18,19]. However, PCa cells can also metastasize to the lungs, liver, lymph nodes, and brain [20,21]. In a healthy individual, there is a balanced process of bone formation and resorption by osteoblast and osteoclast cells, respectively [22]. In cancer, the fine balance tips toward bone resorption, leading to osteolytic lesions as seen in breast and lung cancer. In contrast, PCa cells metastasized to the bone can present with both osteoblastic and osteolytic lesions, osteoblastic lesions being much more common [23,24,25]. Among other mechanisms, the abnormal bone formation is largely driven by PCa cells, which secrete endothelin 1, thus activating Wnt signaling and fibroblast growth factor receptor [26,27]. These two processes lead to reduced skeletal strength and increased risk of fracture development [28,29]. There is evidence to suggest that the presence of DCCs in the bone is a poor prognostic factor [30,31]. Interestingly, however, DCCs do not always lead to overt metastases [32]. Cancer-induced bone changes seem to take place when DCCs reactivate and during the proliferation period. Specifically, reactivated DCCs can lead to the differentiation of osteoclastic precursors and fuel the process of osteolytic metastasis [33,34,35]. In contrast, the interplay between dormant cells and cellular components of bone appears to favor osteoclasts and osteoblasts working in a balanced manner, similar to that of a healthy individual. This suggests that the microenvironment or niche in the secondary organ has a key role in deciding the fate of DCCs [36,37,38,39]. Below we discuss selected literature on the metastatic niche and signaling pathways associated with cellular dormancy, specific to bone marrow (BM), that control the fate of dormant DCCs (Figure 1).

## 3. Niche Cell Types and Signals that Induce or Block PCa DCC Dormancy in the Bone Marrow Microenvironment

Bone marrow is one of the richest and most diverse organs in terms of its cellular and biochemical composition. It contains numerous cell types, such as hematopoietic stem cells (HSCs), mesenchymal stem cells (MSCs), endothelial cells, macroph ages, osteoblasts, osteoclasts, immune cells, stromal cells, megakaryocytes, fibroblasts, and adipocytes [22,23,24,40,41,42,43]. This cellular milieu appears to provide an environment for DCCs to reside in a dormant state. Importantly, understanding HSC dormancy and expansion may provide key insights into the mechanisms of initiation and maintenance of cancer cell dormancy. HSCs can be found close to the arteriolar niche and in sinusoids in BM. In the vicinity lie endothelial cells, MSCs, perivascular stromal cells, reticular cells, and nestin+ MSCs that secrete stem cell factor (SCF) and CXCL12 (C-X-C motif chemokine ligand 12) chemokines that regulate the HSCs behavior [44,45,46,47]. Specifically, CXCL12/CXCR4 chemokine signaling aids in HSC retention in BM, quiescence, and proliferation [48,49,50]. Similarly, the sympathetic nervous system, which functions through the expression of CXCL12 in perivascular cells and osteoblasts, has also been recently reported to regulate DCCs in addition to HSCs [51]. It is believed that sinusoids may provide a proliferative niche to HSCs, because the blockade of E-selectin protects HSCs from chemotherapy [52]. On the other hand, the arteriolar niche, which is distinctly surrounded by sympathetic nerves and layers of smooth muscle cells, maintains HSCs in a dormant and quiescent stage. It was shown that the conditional deletion of CXCL12 does not affect the number of HSCs in BM but induces their mobilization [53].

How are the above mechanisms linked to PCa dormancy and metastasis? CXCL12 is important for PCa cells metastasis in bone [54]. In an in vivo study in a mouse model, it was shown that CXCL12 strongly co-localizes with PCa cells in the metaphysis of long bones, and blocking of CXCL12 receptor CXCR4 with a neutralizing antibody inhibits the homing of PCa cells [55]. Thus, it does not appear that all signals that control HSC quiescence may induce PCa dormancy. CXCR4 also promotes invasion and metastasis in breast cancer (BCa) and PCa through its effector protein phosphatidylinositol 4-kinase IIIa (PI4KIIIα) [56,57]. Additionally, several other factors, such as α5 and β3 integrins expressed by PCa cells and WISP1 (WNT1 induced secreted protein 1) expressed by osteoblasts at the local microenvironment, help in the homing of PCa by facilitating their adhesion and anchorage to the extracellular matrix of the bone [58,59]. WISP1 regulates the expression of VCAM-1 (vascular cell adhesion protein 1) in PCa cells and integrin α4β1 in osteoblasts, as pre-treatment of PCa cells with VCAM-1 antibody or α4β1 antibody to osteoblasts attenuates the capacity of PCa cell adherence to osteoblasts [58]. Even though the role of CXCL12 appears to be important for DCC homing in the bone marrow, the exact location of PCa DCCs in it requires further study. Thus, more work on PCa is needed to understand the location and niche-dependent regulation of dormancy of DCCs.

What are some of the intracellular pathways that might control PCa DCC dormancy? Dormant PCa cells may depend on a well-studied mechanism of dormancy, where the ratio between the p38-MAPK stress response signaling pathway and extracellular signal-regulated kinase (ERK) dictates whether cancer cells proliferate or enter a phase of dormancy [60]. p38 is a negative regulator of ERK, and its higher activation causes G0-G1 arrest, leading cells into quiescence [61,62,63,64]. Transforming growth factor-β2 (TGFβ2), for example, can prompt cellular dormancy by inducing p38^high^/ERK^low^ signaling via Smad1/5, which is also secreted by cells in BM [38,65]. Interestingly, analysis of PCa DCC transcriptomes from patients or analysis of PCa PDX models revealed that patients that have no evidence of disease carry DCCs with a signature reflecting TGFβ2 and p38 pathway activation [66,67], while TGFβ2 can also be pro-quiescence for PCa cells [68]. In a recent study on the BCa model, direct proof was provided showing that HSC dormancy niches control BCa DCC dormancy. Periarteriolar BM-resident NG2+/nestin+ MSCs guide BCa DCCs to enter dormancy by producing TGFβ2 and bone morphogenetic protein (BMP7), which activates a quiescence pathway dependent on TGFFBRIII and BMPRII. Depletion of the NG2+/nestin+ MSCs or knockout (KO) of TGFβ2 specifically from NG2+/nestin+ MSCs leads to metastatic outgrowth in BM [33].

Additionally, several bone-secreted factors mainly from osteoblasts, such as DKK3, vasorin, and neogenin, induce dormancy through activating the p38/MAPK signaling pathway, while BMP1 uses an alternative pathway to promote dormancy [69]. Secreted factors from differentiated osteoblast cells, such as TGFβ2 and GDF10, induce tumor dormancy through activation of the TGFβRIII-p38MAPK-phospho (S249/T252)-retinoblastoma signaling pathway [34,69,70]. Activated phospho-p38MAPK phosphorylates retinoblastoma at the N-terminal S249/T252 sites to block prostate cancer cell proliferation, while the expression of dominant-negative p38MAPK prostate cancer cell lines (C4-2b and C4-2B4) abate cancer cell dormancy [34]. Annexin A2 secreted in the endosteal niche was shown to induce dormancy by upregulating the growth arrest-specific 6 (GAS6) ligands [71,72]. Osteoblasts can also produce GAS6 that prevents the proliferation of human PCa cell line PC3 under in vitro conditions [72]. In another study, it was shown that GAS6 and AXL (from cancer cells) are required for TGFβ2-mediated cell growth suppression in PCa, where AXL positively regulates the expression of TGFβ and TGFβ receptor 2 (TGFβR2). In the PC3 PCa cell line, TGFβ2 but not TGFβ1 could induce GAS6 and p27 expression to regulated dormancy [73]. Thus, PCa DCCs can be programmed into a dormant state by similar mechanisms, as revealed in other bone-seeding cancers that undergo dormancy.

Wnt signaling is another pathway that may be important for dormancy induction. Ren et al. demonstrated that higher expression of Wnt5a in osteoblastic niche induces dormancy of PCa cells by activating non-canonical ROR2/SIAH2 signaling, which results in the inhibition of canonical Wnt/β-catenin signaling [74]. Previously, it was shown that the suppression of Wnt/β-catenin signaling is an early event in the induction and maintenance of dormancy in cancer cells [9]. A decrease in the expression of Wnt5a with aging directly correlates with a higher metastatic burden [75]. Thus, multiple signals from the niche seem to cooperate to induce and maintain PCa dormancy. The multifactorial nature of this process may explain why the process of clinical dormancy can last many years to decades.

Signals that allow cancer cells to switch out of dormancy are also very important. In bone marrow, oxygen tension fluctuates between approximately <1 and –6%, which provides an important signal with varying responses from cancer cells including resistance to therapy [76,77,78]. However, hypoxia may also promote metastasis of DCCs in the bone. Cox et al. showed that under hypoxic conditions, BCa cells secrete lysyl oxidase (LOX), which is a pro-invasive and pro-metastasis factor that is associated with bone tropism and relapse. LOX can drive osteoclastogenesis through NFATc1 that subsequently alters the bone homeostasis and provides a premetastatic niche for disseminated tumor cells [79]. Another study suggests that HIF1α signaling may also drive bone metastasis by activating the expression of DUSP1 and CXCR4 genes [80]. Hypoxia could also promote metastasis by altering the expression of dormancy maintenance genes, such as the leukemia inhibitory factor receptor (LIFR) [81]. This has been shown in BCa MCF7 and SUM159, where hypoxic conditions (<0.5% pO2) negatively regulate LIFR expression. Thus, PCa DCCs in bone marrow that escape dormancy via a niche-dependent control may respond to hypoxia via a reactivation phenotype.

Other changes in the microenvironment of tissues harboring DCCs may also cause reactivation or escape of dormant cancer cells from dormancy, leading to overt metastasis [7,82]. One of the earliest studied aspects of the induction and reactivation of dormant cancer cells was the extracellular matrix (ECM), and it was shown that the association of the urokinase plasminogen activator receptor (uPAR) with α5β1 integrin activates the MEK/ERK pathway and plays an inhibitory role in cancer dormancy [83]. Bone marrow-derived cells, such as tumor-associated macrophages (TAMs) and tumor-associated neutrophils (TANs), produce ECM remodeling proteases in the tumor and release them in the metastatic microenvironment, which promotes tumor angiogenesis [41,84,85]. Vascular endothelial cells remodel the ECM through binding to the integrin receptor that enhances the formation of new vessels [86]. Ghajar et al. showed that metastatic cancer cells are often associated with the basement membrane close to a vascular niche. DCCs residing in perivascular niches, which are maintained in a dormant state by endothelial-derived thrombospondin-1, can exit dormancy because of sprouting neovasculature. These sprouting neovasculatures not only promote the exit of dormant cancer cells but also accelerate tumor growth by secreting TGFβ1 and periostin, which are tumor-promoting factors [86]. The process of osteoclastic bone resorption by osteoclasts, which leads to bone remodeling that changes the cellular composition and signaling, can cause the exit of cancer cells from a dormant state. Receptor activation of NF-kB ligand (RANKL), expressed by PCa cells, establishes a pre-metastatic niche by activating transcription factors, which control EMT, stemness, and osteomimicry [87]. It was shown that inhibiting RANK or its downstream signaling network of c-Myc/Max or c-Met reduced or eliminated skeletal metastasis in mice [88]. Cancer cells themselves induce the osteoclastic bone resorption by secreting factors, such as parathyroid hormone-related protein (PTHRP), which in turn secrete TGFβ1 that further induces PTHRP secretion. This feed-forward cycle also known as the “vicious cycle” accelerates osteoclastic bone resorption [25].

In addition to changes in the microenvironment, alterations in the immune escape of cancer cells could also lead to the exit from dormancy. DCCs in BM downregulate MHC class I, which is required for the detection by CD8+ T cells [89,90]. Dormant PCa cells in the bone are enriched in tumor-intrinsic IFN signaling, which regulates dormancy status and bone remodeling processes through immune activation, leading to a longer bone metastasis-free survival. Loss of tumor intrinsic type I IFN signaling in PCa cells in BM leads to bone metastasis. This loss of intrinsic IFN signaling can be rescued by epigenetic modification through histone deacetylase inhibitors (HDACi) [91]. These studies highlight the complexity of the mechanisms for dormancy onset and reawakening and provide insight into the signals that may present as a good target to prevent reactivation or to induce and maintain dormancy of PCa.

## 4. Therapeutic Approaches Targeting Bone–Tumor Microenvironment

The bone microenvironment serves as fertile soil for the seeding of tumor cells and acts as a potential driver of the metastatic process. Thus, targeting the pathways regulating the BM niche is a promising strategy to prevent and, potentially, treat bone metastases. Anti-resorptive agents, which include bisphosphonates and RANKL inhibitors, were the first bone modifying agents to be clinically tested for possible prevention of bone metastases. This stems from their ability to inhibit osteoclastic activity, a key component for tumor cell seeding, survival, and metastasis activation. Although several studies of bisphosphonates failed to demonstrate a delay in time to bone metastases [92,93,94], denosumab, compared to placebo, did delay the onset of bone metastasis in castration-resistant prostate cancer with non-bone-only metastases (HR 0.85, 95% CI 0.73–0.98, *p* = 0.028) [95]. Despite this evidence, the clinical significance of an observed 4-month delay in bone metastasis remains unknown. As a result, denosumab is not used in clinical practice for the prevention of bone metastases. One major reason for the lack of clinical activity in bone metastasis prevention is that bisphosphonates and denosumab target bone metastases at a later stage and do not seem to influence earlier events in this process.

Advances in our mechanistic understanding of dormancy provide a guide to disrupt bone and, potentially, visceral metastasis formation at an earlier stage. In mouse models of disseminated estrogen-receptor-expressing (ER+) breast cancer in BM, the combination of D9, a thioredoxin reductase inhibitor, with the pan-AKT inhibitorMK-2206 prevented the formation of new metastases better than tamoxifen [96]. Additionally, AKT isoforms are implicated in metastatic outgrowth from lung DCCs [97]. Thus, AKT inhibition is a feasible strategy that needs further exploration. Another potential pathway, currently in early clinical development, is the inhibition of colony-stimulating factor receptor 1 (CSF-1R), which controls macrophage differentiation and alters the bone–tumor microenvironment (NCT02472275, NCT02265536). These mechanisms provide opportunities most likely to target already active metastatic disease, but new approaches might be needed to induce or maintain dormancy at earlier stages.

Since dormancy is a reversible process, epigenetic changes play an important role in regulating the initiation, maintenance, and metastatic reactivation from a dormant state. The epigenetic changes are niche specific, and they may over-ride the metastatic potential of oncogenes and keep the DCCs dormant for a longer period under the control of favorable dormancy cues [98,99,100]. NR2F1, a transcription factor and central regulator of dormancy, causes strong epigenetic changes associated with PCa lineage plasticity and influences response to standard therapies, such as antiandrogens and chemotherapy. When comparing the BM of post-radical prostatectomy patients with no evidence of disease (NED-dormant disease) to advanced proliferative disease (ADV), it was observed that 42.8% of NED DCCs showed NR2F1 upregulation compared to only 10.3% in DCCs derived from ADV patients [66]. In PCa and head and neck squamous cell carcinoma (HNSCC) models, NR2F1 induced a dormant phenotype by regulating the H3K4me3 and H3K27me3 epigenetic marks in the promoter of SOX9 and RARβ. In the same study, cancer cells, including PC3 PCa treated with 5-azacitidine (AZA, an FDA-approved drug), and all-trans retinoic acid (atRA) upregulated dormancy genes, including NR2F1, SOX9, RARβ, and p21, and downregulated Ki67 and P-ERK1/2. The induction of NR2F1 by AZA + atRA led to reprogramming of the epigenetic landscape of cancer cells, and the treatment caused a global increase in the level of the repressive chromatin state. This was demonstrated by enrichment in H3K9me3 and H3K27me3, while the promoter of the dormancy genes became more open. In the in vivo model system, the combination of AZA and atRA induced quiescence for an enduring period beyond the treatment phase. Based on these exciting results, a clinical trial was developed to test the combination of AZA and atRA in biochemically recurrent prostate cancer (NCT03572387). Such approaches may provide opportunities to prevent expanding PCa metastasis, and the latter clinical trial may provide answers as to whether this is a possible approach.

## 5. Conclusions

We have made significant progress in understanding the role of the microenvironment in metastatic progression and dormancy of DCCs since the first time the “seed and soil” hypothesis was postulated by Paget in 1889 [101]. In the context of dormancy of PCa cells, we are yet to explore and broaden our understanding of (a) which signatures and cues provided by the cancer cells at the distant organs are important for dormancy crosstalk with native resident cells; (b) how early cancer cells disseminate to distant sites; (c) what type of cellular and epigenetic reprogramming favors dormancy onset; and (d) whether localization of DCCs in different regions in BM has a role in dormancy. Prostate cancer represents a distinct malignancy amenable to dormancy therapeutic targeting based on the availability of early screening methods, relatively slow growth, and a shortage of effective therapeutic options for advanced disease states. While many foundational questions remain unanswered, available mechanistic knowledge needs to be translated to develop therapeutic targets earlier in the disease course and, ultimately, prevent lethal metastatic disease.

## Data Availability

Not applicable.
